# Does Portuguese pharmaceutical market follow an innovative trend? The INFOMED database analysis

**DOI:** 10.1093/eurpub/ckac131.252

**Published:** 2022-10-25

**Authors:** FG Avelar, B Raposo, A Torres, AR Pedro

**Affiliations:** 1 Public Health Research Centre, National School of Public Health, Lisbon, Portugal; 2 Comprehensive Health Research Centre, Lisbon, Portugal

## Abstract

**Background:**

Product innovation in the pharmaceutical market is, although not exclusively, a new product with the capacity to generate value. Thus, it must represent an invention through investment in research and development, and not a commercial monopoly. The aim is to identify innovation trends by Marketing Authorization (MA) granted in Portugal.

**Methods:**

Quantitative descriptive study based on data (years 2017 to 2021) from Human Medicinal Products Database (INFOMED), managed by the National Authority of Medicines and Health Products, I.P. The parameters identified and used were MA and medical product subject to medical prescription. List was filtered by Product Group - New Active Substance. To identify innovation, findings were compared with the Anatomical Therapeutic Chemical (ATC) database and scientific literature to find other therapeutic options available.

**Results:**

A total of 2695 records were identified. 1804 (67%) were generic drugs. Regarding new substances, 46 registrations (1.7%) were obtained, with the highest number recorded in 2017 (n = 13 - 2.03%) and the lowest in 2019 (n = 3 - 0.76%). After exclusion of different concentrations with the same therapeutic indication, a total of 26 medicines were observed: 54% had a listed ATC code and those, 64% had more than two linked ATC codes; 12 medicines had no related ATC code and 5 were classified as vaccine and therefore were not considered, thus 7 medicines were classified as major innovation.

**Conclusions:**

Most of the innovation seen in the pharmaceutical market is not major innovation, but rather due to structural changes to chemical compositions. The Portuguese scenario is no different. Generic drugs, although not innovative, are important in the pharmaceutical market from a public health perspective. The identified innovations, although not major innovations, are important from a clinical and market availability perspective, however they should not represent a large portion of the pharmaceutical market.

**Key messages:**

• In Portugal, the innovation trend in the pharmaceutical industry is based essentially on modifications in chemical composition and related therapeutic class.

• The development of major innovation in medicines should be stimulated and new models of financing and sustainability should be improved.

